# Relationship between professional identity and academic performance of medical undergraduates in China: the mediating role of self-efficacy

**DOI:** 10.3389/fmed.2025.1597554

**Published:** 2025-11-04

**Authors:** Ge Zhang

**Affiliations:** North Henan Medical University, Xinxiang, China

**Keywords:** professional identity, self-efficacy, academic performance, medical undergraduate, mediating effect

## Abstract

**Introduction:**

Undergraduates’ academic performance is susceptible to psychological factors. This study focused on investigating the relationship between professional identity, self-efficacy and academic performance of medical undergraduate and further explored the mediating role of self-efficacy.

**Methods:**

This study used quantitative research method to collect data from 350 five-year clinical medical undergraduates through an online survey. These participants completed the Professional Identity Questionnaire and the Academic Self-Efficacy Questionnaire, while the researcher assessed their academic performance by counting their GPAs. Correlation analysis was used to explore the relationship between professional identity, self-efficacy, and academic performance, and the mediating effect of self-efficacy was tested through the PROCESS macro of SPSS.

**Results:**

The results showed that there was a positive correlation between medical undergraduates’ professional identity and academic performance, but the correlation between them was weak (*r* = 0.253, *p* < 0.001), and there was a significant positive correlation with self-efficacy of moderate strength (*r* = 0.604, *p* < 0.001). There is a significant positive correlation between self-efficacy and academic performance and the correlation coefficient is greater than 0.7, which is a very strong correlation. Moreover, the mediating effect of self-efficacy between professional identity and academic performance was established and the mediating effect accounted for 85.51% of the total effect.

**Discussion:**

This study contributes to the understanding of the relationship between professional identity, self-efficacy and academic performance of medical undergraduates. There is a correlation between professional identity, self-efficacy and academic performance, but the correlation between self-efficacy and academic performance is the strongest, the correlation between professional identity and self-efficacy is only moderate, and the correlation between professional identity and academic performance is the weakest. Therefore, it is recommended that medical universities and teachers pay great attention to the cognitive and psychological status of medical undergraduates, especially to the mediating role of self-efficacy, so as to help medical undergraduates to improve their academic performance in a targeted way.

## 1 Introduction

As the living standard continues to rise, health has become a growing concern for people, and the demand and requirements for doctors have also increased. In particular, the successive outbreaks of the COVID-19 around the world have posed a serious threat and impact on the physical and mental health of the people, making everyone more aware of the importance of doctors in protecting people’s lives and health, and reinforcing the mission and responsibility of doctors (1). In addition, in the context of the national strategy of “Healthy China,” the all-round and full-cycle maintenance of people’s health has become the core objective, the rapid development of the health service industry, and the rapid advancement of science and technology in the field of health, which have put forward new requirements for changes in the higher medical education in order to cultivate more excellent medical talents (2). According to the Standards for Medical Education in China (the 2022 Revision), the clinical medicine major at the undergraduate education level which is the starting point and the main cradle for the training of doctors carries the important task of training medical graduates with initial clinical ability, lifelong learning ability and excellent professional qualities. Undergraduates majoring in clinical medicine have a broad range of studies covering basic medicine, clinical medicine, preventive medicine, and humanities and social science courses. The integration of multidisciplinary knowledge makes medical undergraduates face great academic challenges (3, 4). As a popular major in medical university, the clinical medicine has a strong attraction to high quality student sources (5). Compared with other non-medical majors, undergraduates who are able to enter the clinical medicine major tend to achieve higher scores in the National College Entrance Examination (NCEE) (6). This reflects the social recognition and expectation of this major, as well as undergraduates’ identification with the major and their aspiration and pursuit of becoming doctors in the future. However, it is alarming that despite their excellent performance in the NCEE, these undergraduates have a pass rate of less than 80% in the Chinese Medical Practitioner Qualification Examination (CMPQE) after 5 years of study (7). As an important measure of medical undergraduates’ academic performance and clinical competence after graduation, the passing rate of the CMPQE directly reflects how many medical undergraduates will become doctors. In other words, more than 20% of medical undergraduates fail to become doctors because of poor academic performance. This data triggers governmental departments, medical universities, and educators to think deeply about the academic performance of medical undergraduates.

Professional identity is a key factor influencing academic performance. Professional identity, as a deep psychological state of an individual toward the major field he or she has studied, is both a static state of being and a dynamic process that evolves with individual experience, cognitive development and changes in the external environment (8). It is not only about the individual’s emotional belonging and acceptance of the current major, but also predicts the individual’s future orientation and attitude in his or her career. It revealed that there is a significant positive correlation between undergraduates’ professional identity and their academic performance (9). Those undergraduates who have a strong sense of identity with their majors are able to make fuller use of these established advantages. They are not only committed to a deep understanding of the inner essence of the subject, but also proactively broaden the boundaries of their learning and constantly explore unknown areas. Such learning attitudes and actions enable them to tend to show greater efficiency and achieve more outstanding results in their academic endeavors. For medical undergraduates, professional identity is unique in that it is not only about personal interest and career development, but also closely related to the knowledge, skills, values, and behaviors necessary for a future career in healthcare (10). Factors such as the mainstream values of the society, the development of the medical industry, the doctor-patient relationship, and the development of one’s own cognition and thinking may have an impact on the professional identity of medical undergraduates (11). Professional identity reflects medical undergraduates’ integration between the norms of the healthcare profession and their personal core values, which positively and positively influences academic performance (12).

Self-efficacy significantly influences academic performance. Self-efficacy refers to an individual’s beliefs about his or her ability to perform a specific task or achieve a specific goal, and it profoundly influences all aspects of human activity by regulating the individual’s cognitive, motivational, affective, and choice processes (13). Individuals with high self-efficacy tend to believe that they have the ability to take control of a situation and act effectively, even in the face of difficulties and challenges (14). In the academic domain, self-efficacy is not only related to undergraduates’ actual abilities, but also largely influences their motivation, strategy choices, and attitudes toward challenges (15). In particular, the level of self-efficacy often becomes an important determinant of academic success or failure when undergraduates are of equal ability. The role of self-efficacy in academic performance has received extensive empirical support. There is a clear positive correlation between academic self-efficacy and academic performance, suggesting that the greater the self-efficacy of undergraduates in their academic abilities, the greater their academic performance tends to be (16). A study of the relationship between academic self-efficacy and academic performance also found that academic self-efficacy had a negative effect in predicting undergraduates’ fear of academic failure, meaning that undergraduates with higher self-efficacy were generally less likely to worry that they would fail academically (17). In addition to this, while the changes in the learning environment brought about by the COVID-19 challenged undergraduates’ academic performance, undergraduates’ self-efficacy did not decrease as a result, suggesting that undergraduates with high self-efficacy were able to maintain their academic performance from being significantly impacted, even in the face of unfavorable external conditions (18). However, it is worth noting that self-efficacy is not static and fluctuates with the stage of study. Medical undergraduates face a five-year long undergraduate study cycle, as well as a heavy and complex study load. As the grade level increases and the difficulty of the course gradually increases, medical undergraduates may encounter more learning barriers and negative emotions, all of which may weaken their self-efficacy, which in turn may adversely affect academic performance (19).

Apart from that, self-efficacy can not only directly influence academic performance, but also indirectly contribute to academic performance through mediation. While self-management may predict undergraduates’ academic achievement to some extent, self-management is certain that undergraduates will achieve successful academic achievement by influencing their self-efficacy, such as confidence in academic tasks and mastery of study skills (20). Self-efficacy can also play a significant mediating role in the relationship between motivation and academic performance of medical undergraduates, and intrinsic motivation of medical undergraduates influences self-efficacy and thus their academic performance (21). Undergraduates’ strong intrinsic achievement motivation can likewise stimulate positive self-efficacy, leading to better academic performance (22). Also, although optimism does not directly affect academic performance, it can affect academic performance through the mediating effect of self-efficacy (23).

In summary, although there have been studies analyzing the relationship with academic performance from the aspects of professional identity and self-efficacy respectively, showing that they do have a certain degree of influence on academic performance, there is no research on the relationship between professional identity, self-efficacy, and academic performance of medical undergraduates majoring in clinical medicine, especially the mechanism of the mediating role of self-efficacy on professional identity and academic performance. Therefore, this study investigates the relationship between professional identity, self-efficacy and academic performance and further explores the mediating role of self-efficacy based on a population of Chinese medical undergraduates majoring in clinical medicine. Based on this, this study proposed four hypotheses.

*H1*: There is a significant correlation between professional identity and academic performance.

*H2*: There is a significant correlation between professional identity and self-efficacy.

*H3*: There is a significant correlation between self-efficacy and academic performance.

*H4*: Self-efficacy mediates the relationship between professional identity and academic performance.

## 2 Materials and methods

### 2.1 Participants and procedure

In order to test the four hypotheses of this study, a quantitative research method was conducted. The study purposefully selected undergraduates from the clinical medicine major at a medical university in central China as the research subjects, as they reflect the lowest pass rate for CMPQE in terms of academic performance. According to the official website of this medical university, there are currently 1,928 undergraduates enrolled in the clinical medicine major. Following a stratified sampling method with a rate of no less than 16% per grade, a total of 350 undergraduates were selected as the sample, comprising undergraduates from the first to the fifth year in order to ensure that the potential interference of study duration on the research results was minimized. All participants volunteered to join the study after being fully informed of its contents and signed an informed consent form before the study began. After obtaining permission from the relevant department of the university, an online questionnaire was used to collect data efficiently, with ample time of 40 to 50 min set aside for each participant to complete the questionnaire. The data collection process lasted for one month from June 2024 onwards. All procedures were conducted in accordance with the ethical principles outlined in the Declaration of Helsinki. Table 1 presents the demographics of the participants.

**Table 1:** Demographic profile of participants.

Variable		Frequencies	Percentage (%)
Gender	Male	182	52
	Female	168	48
Grade	1	72	20.57
	2	64	18.29
	3	64	18.29
	4	70	20
	5	80	22.85
The choice of major comes from	Individual	282	80.57
	Parents	64	18.29
	Others	4	1.14
Whether the family has a medical doctor	Yes	268	76.57
	No	82	23.43

*N* = 350.

### 2.2 Instruments

#### 2.2.1 Professional identity questionnaire

The Professional Identity Questionnaire was used to measure medical undergraduates’ professional identity, which was divided into four dimensions, namely, professional cognitive, professional emotion, professional behavior, and professional fitness, with a total of 23 items (8). The questionnaire is scored on a 5-point scale, with 1 meaning “not at all” and 5 meaning “completely.” The total score was calculated by summing the scores of all its items. A higher total score indicates a higher level of professional identity among the undergraduates. This questionnaire has been proved to be a reliable and valid questionnaire by many studies in China (24–27). In this study, the Cronbach’s *α* of the total questionnaire was 0.967 and the Cronbach’s α of each dimension was 0.919, 0.949, 0.930, and 0.866, which indicated that the questionnaire had good reliability.

#### 2.2.2 Academic self-efficacy questionnaire

Medical undergraduates’ self-efficacy was measured using the Academic Self-Efficacy Questionnaire (ASEQ), which was divided into two separate dimensions of self-efficacy for learning abilities and self-efficacy for learning behaviors, with a total of 22 items (28). The questionnaire is scored on a 5-point scale ranging from “not at all” to “completely.” The total score was calculated by summing the scores of all its items. A higher total score indicates a higher level of self-efficacy among the undergraduates. It has been proven to be a reliable and valid questionnaire in China by many studies (29–32). In this study, the Cronbach’s *α* of the total questionnaire was 0.940 and the Cronbach’s α of each dimension was 0.945 and 0.812, which indicated that the questionnaire had good reliability.

#### 2.2.3 Grade point average (GPA)

Statistics on undergraduates’ GPA to measure academic performance. The advantage of GPA as an indicator of academic performance is its fully quantitative, objective and comparable nature (33). In order to ensure the authenticity and objectivity of the data, their GPA was provided by the academic affairs office of this medical university.

### 2.3 Data analysis

Non-parametric statistical methods were employed to analyze the correlations among professional identity, self-efficacy, and academic performance test hypotheses 1 to 3. Finally, the mediation effect of self-efficacy between professional identity and academic performance was tested using the PROCESS macro (version 3.5) in SPSS, a commonly used mediation analysis method like Structural Equation Modeling (SEM) (34). PROCESS can automatically process the data, which reduces the errors that may be caused by human operation and improves the accuracy and efficiency of the analysis (35). To estimate the 95% confidence interval for the mediating effect in the model, 5,000 bootstrap resamples were generated (36).

## 3 Results

### 3.1 Professional identity and academic performance

Spearman correlation analysis was used to explore the relationship between professional identity and academic performance as well as the strength of the relationship, and the results are shown in Table 2.

**Table 2:** The Correlation between professional identity and academic performance.

Construct	Academic performance	Professional identity
Academic performance	1.000	
Professional identity	0.253^**^	1.000

^**^Correlation is significant at the 0.01 level (2-tailed).

The absolute value of the correlation coefficient is closer to 1, which indicates a strong correlation between variables, and closer to 0, which indicates that there is no relationship (37). A positive correlation coefficient indicates a positive correlation, while a negative correlation coefficient indicates a negative correlation. A correlation coefficient between ±0.1 and ±0.3 indicates a weak correlation (38). Hence, there is a positive correlation between medical undergraduates’ professional identity and academic performance, but the correlation between them is weak (*r* = 0.253, *p* < 0.001).

Therefore, H1 is established that there is a significant correlation between professional identity and academic performance.

### 3.2 Professional identity and self-efficacy

Spearman correlation analysis was used to explore the relationship between professional identity and academic performance as well as the strength of the relationship, and the results are shown in Table 3.

**Table 3:** The correlation between professional identity and self-efficacy.

Construct	Professional identity	Self-efficacy
Professional identity	1.000	
Self-efficacy	0.604^**^	1.000

**Correlation is significant at the 0.01 level (2-tailed).

According to the suggestion of Haldun Akoglu (38), the correlation is moderate strength when the correlation coefficient is more than 0.4 but less than 0.7 (38). As shown in Table 4, there is a significant positive correlation between professional identity and self-efficacy, and the strength of the correlation is moderate (*r* = 0.604, *p* < 0.001).

**Table 4:** The correlation between self-efficacy and academic performance.

Construct	Self-efficacy	Academic performance
Self-efficacy	1.000	
Academic performance	0.893**	1.000

^**^Correlation is significant at the 0.01 level (2-tailed).

Therefore, H3 is established that there is a significant correlation between self-efficacy and academic performance.

Therefore, H2 is established that there is a significant correlation between professional identity and self-efficacy.

### 3.3 Self-efficacy and academic performance

Spearman correlation analysis was used to explore the relationship between self-efficacy and academic performance and the strength of the relationship. As shown in Table 4, there is a significant positive correlation between self-efficacy and academic performance, and the correlation coefficient is greater than 0.7, which is a very strong correlation.

### 3.4 Mediating effect of self-efficacy

This study used the Bootstrap confidence interval to examine the mediating role of self-efficacy between professional identity and academic performance through the PROCESS macro in SPSS. PROCESS macro mediation model proposed the criterion for judging the mediation effect, which is that if the 95% confidence interval (CI) includes zero, fail to reject the null hypothesis (39). The null hypothesis of this study is self-efficacy does not mediate the relationship between professional identity and academic performance. According to Table 5, the 95% CI [0.008, 0.017] for the indirect effect did not contain zero, which indicated that self-efficacy had a mediating effect between professional identity and academic performance (36). And the indirect effect (0.183) accounts for 85.51% of the total effect (0.214).

**Table 5:** Total, direct, and indirect effects for mediation model.

Total, direct, and indirect effects	b	t	95% Confidence interval		*p*	Quantity of effect
			LLCI	ULCI		
Total Effect (PI→AC)	0.214	5.886	0.008	0.016	0.998	100%
Direct Effect (PI→AC)	0.021	0.002	−0.004	0.006	0	14.49%
Indirect Effect (PI→SE → AC)	0.183	0.002	0.008	0.017	0	85.51%

Therefore, H4 is established that self-efficacy mediates the relationship between professional identity and academic performance.

## 4 Discussion

The first hypothesis of this study aimed to verify the correlation between medical undergraduates’ professional identity and academic performance. The results of the study showed that there is a significant positive correlation between medical undergraduates’ professional identity and academic performance, which means that the more medical undergraduates identify with their major, the higher the likelihood of obtaining excellent academic performance. This finding is consistent with the results of several previous studies that have demonstrated that professional identity is a significant predictor of academic performance (9, 40, 41). Undergraduates with a high degree of professional identity have a relatively high level of awareness, emotional acceptance, and persistence in their major subject matter, and are naturally more likely to reap the rewards of more excellent academic performance. The results of the study also revealed that the direct influence of medical undergraduates’ professional identity on academic performance is weak. In other words, professional identity is an influential factor in the academic performance of medical undergraduates, but it is not the only influential factor and has a weak influence. This result is very different from previous studies.

The second hypothesis of this study aimed to verify the correlation between professional identity and self-efficacy of medical undergraduates. The results of the study indicated that there is a significant positive correlation between professional identity and self-efficacy among medical undergraduates. This is consistent with the results of several previous studies (19, 42, 43). Behavioral expression of medical undergraduates is influenced by professional identity, positive self-perception enhances evaluation of their knowledge and competence, and self-efficacy is reflected through individuals’ self-assessment of their own competence, so self-efficacy increases with the level of professional identity (44).

The third hypothesis of this study aimed to verify the correlation between medical undergraduates’ self-efficacy and academic performance. The results of the study showed that there is a significant positive correlation between self-efficacy and academic performance of medical undergraduates and the correlation is strong. This result is similar to the findings of a number of previous studies, all of which confirmed that self-efficacy can positively influence academic performance (18, 20, 45–47). Undergraduates with a strong sense of self-efficacy are more enthusiastic and motivated in learning, and tend to set lofty goals and pursue them diligently. They believe they can master knowledge and skills, see setbacks as opportunities for growth, and maintain a positive mindset to face challenges. This attitude is particularly important in complex disciplines such as medicine, enabling them to calmly cope with clinical practice, find solutions, and enhance their academic performance. In addition, they possess strong time management and self-supervision skills, and are able to rationalize their study time, formulate and execute scientific plans, and identify and solve problems in a timely manner. This ability not only improves learning efficiency, but also ensures the successful completion of academic tasks. Therefore, undergraduates with a strong sense of self-efficacy show more advantages in their studies, which helps them to succeed in their studies.

The last hypothesis of this study aimed to verify the mediating role of medical undergraduates’ self-efficacy between professional identity and academic performance. The findings revealed the mediating role of self-efficacy, operating through the following mechanism: a strong professional identity provides medical students with a profound sense of learning significance and intrinsic motivation. When medical undergraduates perceive themselves as future doctors rather than merely undergraduates preparing for examinations, mastering complex medical knowledge acquires greater value. For instance, the conviction that ‘I must master physiology to save lives in the future’ fosters this sense of meaningfulness. This translates into a steadfast confidence to overcome academic challenges, that is, self-efficacy (48). Subsequently, this robust self-efficacy directly influences their learning behaviors. Students with high self-efficacy tend to set more ambitious learning goals, striving for excellence rather than merely passing (49). They adopt more proactive and in-depth learning strategies, such as actively seeking clinical case connections to theory or organizing peer discussions. When encountering setbacks like exam failures or struggling with complex concepts, they demonstrate greater resilience and persistence. Ultimately, these sustained and efficient learning behaviors directly contribute to improved academic performance. This finding aligns with the conclusions of the study on undergraduates in physical education (50) and the study on nursing undergraduates (51). This indicates that the role of self-efficacy in linking professional motivation to learning outcomes possesses cross-disciplinary universality. However, unlike the finding that professional identity had a direct effect on academic performance (52), this study reveals a more intricate internal mechanism: self-efficacy serves as an indispensable psychological mediator. This divergence may stem from the particularities of the Chinese medical undergraduates’ sample and cultural context, highlighting the pivotal bridging role of psychological beliefs within China’s highly competitive medical education environment.

This study explored the relationship between professional identity, self-efficacy, and academic performance among medical undergraduates and further investigated the mediating role of self-efficacy between professional identity and academic performance. As with all studies, this study has some limitations. First, the sample for this study was drawn from undergraduates majoring in clinical medicine at a medical university in central China. Although the sample size is sufficient to support the results of this study, the representative is limited. The results of this study are useful for other medical universities and medical majors, but cannot be directly used in their studies. Future research on this topic could expand the scope of sample sampling and increase the sample size, such as sampling medical undergraduates from the Eastern, Central, and Western regions of China, respectively, to improve the general applicability of the findings. It can also be based on international research perspectives, take samples from abroad, and conduct comparative studies between China and abroad. Second, the research instruments in this study were not designed for medical undergraduates. Although these two research instruments have been used many times by previous researchers for medical undergraduates (25, 53), the items in them still do not fully reflect the characteristics of the medical major. Future research could incorporate existing research instruments that specialize in medical undergraduates and develop questionnaires on professional identity and self-efficacy, which could be more relevant to the research topic and study population. Finally, this study found a weak, though significant, relationship between professional identity and academic performance. Future research could explore the effects of other factors besides self-efficacy on the relationship between them, such as motivation, learning strategies, and learning engagement. In addition to this, qualitative research methods can be used to explore the predictors of professional identity and self-efficacy.

## 5 Conclusion

Although the government, medical universities and teachers have made many efforts to improve the academic performance of medical undergraduates, many of them fail to meet the requirements of doctors after graduation. The findings indicate that self-efficacy serves as a crucial psychological bridge linking medical undergraduates’ professional identity with their academic performance. And this study also reminds medical educators that strategies for improving academic performance should not be confined solely to optimizing curriculum content and teaching methods, but should instead adopt a dual-pronged approach, focusing equally on medical undergraduates’ psychological and professional development (54–56). For cultivating outstanding medical undergraduates not only imparting knowledge but also forging their noble professional identity and robust professional confidence. Future educational practices should integrate psychological interventions with academic guidance to achieve medical undergraduates’ holistic development.

## Data Availability

The raw data supporting the conclusions of this article will be made available by the authors, without undue reservation.
